# The Cellular and Immune Context of YTHDC1 in Hepatocellular Carcinoma: A Single‐Cell and Multicohort Study

**DOI:** 10.1155/ijog/6542992

**Published:** 2026-10-10

**Authors:** Siqi Luo, Diguang Wen, Jingyi Zhang, Zuojin Liu, Jiao Lu, Jianchuan Deng, Hanqing Zeng, Shifeng Lou, Yue Li, Lin Lv

**Affiliations:** ^1^ Department of Hematology The Second Affiliated Hospital of Chongqing Medical University Chongqing China cqmu.edu.cn; ^2^ Department of Thoracic Surgery University-Town Hospital of Chongqing Medical University Chongqing China cqmu.edu.cn; ^3^ Department of Hepatobiliary Surgery The Second Affiliated Hospital of Chongqing Medical University Chongqing China cqmu.edu.cn; ^4^ Department of Gastroenterology The Second Affiliated Hospital of Chongqing Medical University Chongqing China cqmu.edu.cn

**Keywords:** hepatocellular carcinoma, immune checkpoint inhibitor, macrophage, multicohort analysis, single-cell RNA sequencing, tumor immune microenvironment, YTHDC1

## Abstract

**Background:**

YTHDC1 is a nuclear N6‐methyladenosine reader involved in RNA processing. Its abundance, cellular distribution, and relationship with the immune microenvironment in hepatocellular carcinoma (HCC) remain incompletely characterized.

**Methods:**

We integrated paired tumor–adjacent transcriptomic and proteomic cohorts, three single‐cell RNA‐sequencing datasets containing 249,080 quality‐controlled cells, four outcome cohorts, three immune checkpoint inhibitor (ICI) cohorts, paired clinical specimens, and cell‐based YTHDC1‐knockdown assays. Patient‐level analyses covered hepatocyte programs, immune states, expression‐supported ligand–receptor axes, survival, ICI response, and exploratory single‐cell network perturbation.

**Results:**

YTHDC1 protein abundance was higher in 160 paired CPTAC samples (Hedges′ *g* = 1.21, 95% confidence interval [CI] 1.01–1.42), whereas RNA effects varied across cohorts. Single‐cell analysis localized YTHDC1 across hepatic and immune compartments and identified patient‐level associations with hepatocyte, lymphoid, and myeloid programs. SPP1–CD44 received the strongest cross‐cohort expression support among prespecified hepatocyte‐to‐myeloid axes. A transferred hepatocyte state showed a pooled tumor–adjacent effect of *g* = 0.42 (95% CI 0.04–0.80), and higher YTHDC1 abundance was associated with poorer overall survival in study‐aware univariable models (hazard ratio 1.20, 95% CI 1.06–1.36). In 37 ICI‐treated patients, the pooled responder–nonresponder effect for YTHDC1 was small (*g* = 0.05, 95% CI −0.60 to 0.71). Exploratory virtual knockout showed modest cross‐cohort agreement in network perturbation ranks. Paired clinical tissues supported increased YTHDC1 mRNA and protein abundance, and YTHDC1 silencing reduced proliferation‐related readouts and THP‐1 migration in two HCC cell lines.

**Conclusions:**

YTHDC1 is increased at the protein level in HCC and is situated within identifiable hepatocyte and immune programs. Functional assays support its contribution to HCC cell proliferation and tumor cell–dependent macrophage‐like cell migration under the tested conditions.

## 1. Introduction

Liver cancer remains a leading cause of cancer‐related death worldwide, and hepatocellular carcinoma (HCC) accounts for most primary liver malignancies [1, 2]. Curative resection, transplantation, and local ablation are available to selected patients, whereas transarterial treatments and systemic therapies are used for more advanced disease [2, 3]. Atezolizumab plus bevacizumab and tremelimumab plus durvalumab have expanded first‐line treatment options for unresectable HCC [4, 5]. Even so, many patients present with advanced tumors or impaired liver function; durable benefit from immune checkpoint inhibitors (ICIs) is confined to a subset, and validated molecular markers are not routinely used to select an ICI‐containing regimen [6, 7]. Defining tumor cell programs together with their surrounding immune context may, therefore, help refine the biological interpretation of HCC and guide biomarker development.

N6‐methyladenosine (m6A) is a prevalent internal RNA modification controlled by writer, eraser, and reader proteins [8]. YTHDC1 is a nuclear m6A reader that regulates pre‐mRNA splicing [9] and promotes the nuclear export of methylated transcripts [10]. Through these functions, YTHDC1 may influence RNA processing, cell state maintenance, and tumor behavior. YTH domain proteins have been associated with prognosis and immune infiltration in HCC [11], and KIAA1429‐mediated suppression of RND3 was reported to involve YTHDC1 during HCC cell metastasis [12]. These findings make YTHDC1 biologically relevant, although its abundance across RNA and protein platforms and its distribution among hepatic and immune compartments require broader evaluation.

Single‐cell RNA sequencing can resolve the cellular ecosystems of primary and metastatic HCC [13] and distinguish diverse infiltrating lymphocyte states [14]. Longitudinal single‐cell studies further show that malignant, stromal, and immune programs change during therapy [15, 16]. Integration of these data with bulk transcriptomics, proteomics, and clinical outcomes can connect cell‐resolved observations to larger patient populations [17–19]. This strategy also requires careful control of the inferential unit: patient‐level replication should support single‐cell comparisons [20], cohort structure should be retained during synthesis, and gene‐associated states should be examined with specificity and sensitivity analyses.

In this study, we examined YTHDC1 across paired bulk RNA and protein cohorts, three HCC single‐cell datasets, survival cohorts, ICI‐treated cohorts, paired clinical tissues, and cell‐based perturbation experiments. We first established tissue‐level abundance and cellular localization and then evaluated YTHDC1‐associated hepatocyte and immune programs, myeloid states, ligand–receptor expression support, cross‐platform state transfer, clinical outcomes, and an exploratory network virtual knockout. Finally, we related these public data findings to YTHDC1 expression in paired clinical samples and to proliferation and macrophage‐like cell migration after YTHDC1 silencing.

## 2. Materials and Methods

### 2.1. Public Datasets and Analysis Populations

TCGA‐LIHC molecular and clinical data were obtained through the Genomic Data Commons and UCSC Xena [21–23], ICGC LIRI‐JP data from Release 28 [24], and CPTAC HCC proteomics from Proteomic Data Commons study PDC000198 [25]. GEO provided GSE14520, GSE149614, GSE290925, GSE112271, GSE202069, GSE215011, and GSE279750 [26]; accession‐linked reports were used for GSE14520 [27], GSE149614 [13], GSE112271 [28], GSE202069 [29], and GSE215011 [30]. TCGA‐LIHC included 371 tumors and 50 nontumor tissues, with 50 matched pairs and 365 patients with overall‐survival data (131 deaths). ICGC LIRI‐JP included 231 tumors and 194 nontumor tissues, with 194 matched pairs and 231 patients with overall‐survival data (42 deaths). GSE14520 comprised 225 tumors and 220 adjacent tissues on GPL3921 (214 pairs; 221 patients with survival data) and 22 tumors and 21 adjacent tissues on GPL571 (19 pairs; 21 patients with survival data). An overlap audit of 231 and 22 unique patient identifiers on GPL3921 and GPL571, respectively, found no overlap between platforms. CPTAC included 165 tumor and 165 nontumor protein profiles, with 160 matched pairs. Single‐cell inputs comprised 71,915 cells from 21 specimens and 10 patients in GSE149614, 149,021 cells from 12 patients in GSE290925, and 49,674 cells from seven specimens and two patients in GSE112271. ICI response was available for 17 patients in GSE202069, 10 in GSE215011, and 10 in GSE279750. The patient was the inferential unit for bulk, survival, ICI, and inference‐eligible single‐cell analyses. GSE112271 was used descriptively because its specimens represented only two patients. Matrices from different platforms were analyzed separately.

The public datasets and primary analysis units are summarized in Table 1.

**Table 1:** Public datasets and primary analysis units.

Resource	Data layer and analyzed population	Primary analysis unit
TCGA‐LIHC	RNA: 50 matched tumor–nontumor pairs; overall survival: 365 patients	Patient
ICGC LIRI‐JP	RNA: 194 matched pairs; overall survival: 231 patients	Patient
GSE14520 GPL3921/GPL571	RNA: 214 and 19 matched pairs; survival: 221 and 21 patients	Patient within platform; one combined GSE14520 study contribution
CPTAC PDC000198	Protein: 160 matched tumor–nontumor pairs	Patient
GSE149614	Single‐cell RNA sequencing: 71,547 quality‐controlled cells from 10 patients	Patient
GSE290925	Single‐cell RNA sequencing: 142,735 quality‐controlled cells from 12 patients	Patient
GSE112271	Single‐cell RNA sequencing: 34,798 quality‐controlled cells from two patients	Descriptive patient/specimen summary
GSE202069, GSE215011, and GSE279750	Pretreatment ICI expression and response: 17, 10, and 10 patients	Patient within cohort

### 2.2. Bulk Transcriptomic, Proteomic, and Clinicopathological Analyses

YTHDC1 was mapped to Ensembl ENSG00000083896. TCGA‐LIHC expression was analyzed from the GDC/Xena STAR‐normalized matrix on the source log2 (count +1) scale [21, 23], with sample‐type codes 01 and 11 identifying primary tumor and solid tissue normal samples. ICGC LIRI‐JP Release 28 FPKM values were transformed as log2 (FPKM +1) [24]. GSE14520 GPL3921 and GPL571 were processed separately on their log2 RMA scales [27]. Affymetrix probes 212455_at and 214814_at represented YTHDC1; their mean was used for the primary estimate, and the individual probes were retained for sensitivity analysis. Healthy donor samples were excluded from adjacent tissue contrasts. CPTAC PDC000198 TMT11 relative abundance was analyzed on its source log2‐ratio scale after aliquot, case, and tissue category reconciliation [25]. Multiple observations from one patient and tissue group were collapsed to their median. No cross‐platform batch correction was applied. Paired tumor–adjacent effects were calculated within each data stratum. Clinicopathological analyses in TCGA and ICGC grouped pathological stage as I–II versus III–IV and grade as 1–2 versus 3–4, analyzed age continuously, and retained sex as recorded in the source data.

### 2.3. Single‐Cell Quality Control, Harmonization, and Atlas Construction

Raw count matrices from GSE149614, GSE290925, and GSE112271 were processed independently with Python 3.13.9 (NumPy 2.4.4, pandas 2.3.3, and SciPy 1.17.1) and R 4.5.1. Cells were retained when they contained at least 200 detected genes, at least 500 total counts, no more than 25% mitochondrial transcripts, and a detected gene count at or below the specimen‐specific 99.5th percentile. Expression was normalized as log [1 + (gene count/total count) × 10,000]. Published labels were harmonized for GSE149614 [13]. In GSE290925 and GSE112271, cells were assigned to the highest‐scoring hepatocyte, T/NK cell, B‐cell, myeloid, endothelial, or fibroblast marker program; cells with a maximum score below 0.08 were labeled “Other.” After quality control, 71,547, 142,735, and 34,798 cells were retained, respectively. A shared‐feature atlas used 427 genes present across all datasets. Cells were balanced by specimen and cell class with seed 20260717 to form a 38,281‐cell visualization subset, expression was centered within cohort, and t‐distributed stochastic neighbor embedding was used for visualization. Marker support, tissue source, cohort, patient composition, YTHDC1 expression, and YTHDC1 detection were displayed on the same atlas. Patient‐level summaries used the complete post–quality control matrices rather than the visualization subset. Seurat 5.4.0 supported single‐cell data handling and marker‐based summaries [31].

### 2.4. Tumor Sample Hepatocytes and Copy Number Variation (CNV) Sensitivity Analysis

Hepatocytes from tumor samples were defined by lineage annotation and specimen source without assuming malignant identity. In GSE149614, published hepatocyte labels from tumor or portal vein tumor thrombus specimens formed the tumor sample compartment, whereas hepatocytes from normal or adjacent tissue provided the nontumor context. In GSE290925 and GSE112271, hepatocyte‐labeled cells from tumor specimens were retained when either the epithelial/tumor‐associated marker score (GPC3, AFP, EPCAM, KRT8, KRT18, and KRT19) exceeded 0.04, or the liver identity score (ALB, APOA1, APOA2, TTR, and ASGR1) exceeded 0.25. YTHDC1 expression and detection were aggregated by patient or patient tissue record. Expression‐inferred CNV was evaluated as a sensitivity analysis in GSE149614 patients with at least 50 post–quality control hepatocytes from both normal and tumor samples. Up to 800 cells per patient and tissue were sampled with seed 20260829. Genes were positioned using GENCODE v38, sex chromosomes were excluded, counts were normalized to 10,000 and log‐transformed, and infercnvpy 0.4.5 was run separately for each patient using that patient′s normal hepatocytes as the reference (window 100 genes, Step 10, log‐fold‐change clip 3, dynamic threshold 1.5). The per‐cell CNV score was the mean absolute inferred signal. Patient‐level median differences and the fraction of tumor sample cells above the within‐patient normal 95th percentile were summarized.

### 2.5. Patient‐Blocked Differential Expression and Hepatocyte Programs

Within each eligible patient, tumor sample hepatocytes were divided at the patient‐specific median of depth‐residualized YTHDC1, and a high–low comparison was retained only when both groups contained at least 20 cells. Raw counts were summed within patient state bins, yielding paired pseudobulk profiles for 10 GSE149614 patients and 11 of 12 GSE290925 patients. Differential expression was performed separately in each cohort with edgeR 4.6.3 using filterByExpr, trimmed‐mean‐of‐*M*‐values normalization, robust dispersion estimation, and a robust quasi‐likelihood negative binomial model [32, 33]. The design was ~ patient + state, and the state coefficient represented YTHDC1‐high minus YTHDC1‐low. Transferable genes were required to have a positive effect in both cohorts. Competitive rank‐based enrichment against Hallmark, Reactome, and Gene Ontology sets used two‐sided Mann–Whitney tests and Benjamini–Hochberg adjustment without directional pathway selection. Proliferation (MKI67, PCNA, TOP2A, CDK1, CCNB1, CCNB2, and MCM2–MCM7), E2F (MCM2–MCM7, CDC6, CDK1, CCNE1, and E2F1), and MYC (MYC, NPM1, NCL, EIF4E, LDHA, ENO1, PKM, and RPLP0) programs were evaluated among patients with at least 80 tumor sample hepatocytes. In the sensitivity analysis, YTHDC1 and each program score were residualized against log1p (total counts), log1p (detected genes), and mitochondrial percentage within patient or specimen. Leave‐one‐patient‐block‐out pathway rankings were used to evaluate enrichment stability.

### 2.6. Lymphoid and Broader Immune Context Analysis

The immune atlas included T/NK, B, and myeloid cells from the post–quality control datasets. Canonical annotation scores, patient composition, paired tissue composition where available, YTHDC1 expression, and detection fraction were calculated within each immune compartment. Functional programs for cytotoxicity, T‐cell activation, interferon response, inflammatory activity, and myeloid suppression, together with selected immune checkpoint and interferon interface genes, were summarized at the patient level. Associations between mean YTHDC1 expression in tumor sample hepatocytes and immune cell abundance or program scores were evaluated separately in GSE149614 and GSE290925 using Spearman′s correlation. Approximate 95% confidence intervals were obtained by Fisher transformation when possible, and direction across cohorts was recorded without merging cell‐level observations.

### 2.7. Myeloid State Analysis

Post–quality control myeloid cells were assigned to the highest scoring of five expression programs: C1QC/FOLR2 (C1QA, C1QB, C1QC, FOLR2, and APOE), FCN1 monocyte (FCN1, S100A8, S100A9, CTSS, and LILRB1), inflammatory (IL1B, CXCL8, TNF, NFKBIA, and PTGS2), interferon (ISG15, IFIT1, IFIT3, CXCL10, and STAT1), or SPP1/TREM2 (SPP1, TREM2, GPNMB, LGALS3, and MARCO). Marker support, patient‐specific state fractions, paired tissue distributions, YTHDC1 abundance across states, and expression of SPP1/TREM2‐related genes were examined. For each patient, total myeloid fraction, state fractions, and mean chemotaxis (CCL2, CCL3, CCL4, CCL5, CXCL8, CXCL10, CXCL12, and CSF1), suppressive myeloid (CD274, LGALS9, TGFB1, IL10, ARG1, MRC1, and VSIR), inflammatory, and interferon scores were calculated. Associations with mean YTHDC1 expression in tumor sample hepatocytes were tested by Spearman′s correlation in GSE149614 and GSE290925. *p* values were adjusted across the displayed myeloid context family by the Benjamini–Hochberg method, and minimum‐cell thresholds were varied in sensitivity analyses.

### 2.8. Expression‐Supported Ligand–Receptor Analysis

Twelve prespecified hepatocyte‐to‐myeloid ligand–receptor axes were evaluated: CCL2–CCR2, CCL3–CCR5, CCL4–CCR5, CCL5–CCR5, CXCL8–CXCR1, CXCL8–CXCR2, CXCL10–CXCR3, CXCL12–CXCR4, CSF1–CSF1R, SPP1–CD44, LGALS9–HAVCR2, and TGFB1–TGFBR1/TGFBR2. Ligand expression in tumor sample hepatocytes and receptor expression in myeloid cells were summarized for each patient. The expression support score was √[max (ligand mean, 0) × max (receptor mean, 0)]; the receptor term for TGFB1 was the mean of TGFBR1 and TGFBR2. CellChat 1.6.1 and CellChatDB.human were used to confirm nomenclature and database membership [34]. Communication probabilities were not estimated. Cross‐cohort priority was calculated as the rescaled median score × [0.5 + 0.5 × directional concordance]. Associations involving YTHDC1, axis scores, chemotaxis, and receiver cell programs were adjusted within separate Benjamini–Hochberg families. Leave‐one‐patient‐out rankings were calculated to assess the stability of axis prioritization.

### 2.9. Transfer of the YTHDC1‐High Hepatocyte State to Bulk Cohorts

A 24‐gene state was defined before bulk projection from genes with a positive YTHDC1‐high‐versus‐YTHDC1‐low pseudobulk direction in both GSE149614 and GSE290925. Explicit immune, erythroid, stromal, and endothelial markers were excluded. The state comprised YTHDC1, TMEM200C, CNTD1, KRT80, TAF4B, CHRNA4, CLDN9, DIRAS2, SCUBE3, SCGN, ZNF615, ZNF454, ZNF418, DLG3, OXTR, MOGAT2, TOX3, AKR7A3, DKK1, CYP39A1, KIF14, CDCA2, KIF20A, and PLK1; the last four genes were designated proliferation linked. The state was projected independently into TCGA‐LIHC, ICGC LIRI‐JP, GSE14520‐GPL3921, and GSE14520‐GPL571. Expression was standardized gene by gene within each stratum, and the score was the mean of available standardized values. For multiple represented microarray genes, the probe with the largest interquartile range was used for the state score; direct GSE14520 analysis of YTHDC1 used the verified mean of probes 212455_at and 214814_at. AnnotationDBi 1.70.0 and org.Hs.eg.db supported identifier mapping. Specificity branches removed YTHDC1, removed the four proliferation‐linked genes, or applied the same pipeline to a fixed 20‐gene comparator set (RAB5A, RAB7A, TBP, POLR2A, PPIA, PSMB2, PSMB4, GUSB, HMBS, PGK1, SDHA, VCP, COPS5, EIF2S1, SEC61A1, ARF1, CAPZA1, CSNK2A1, NONO, and SNRPD3). No outcome‐derived optimization or threshold was used.

### 2.10. Study‐Aware Tissue Synthesis and Survival Analysis

Paired tissue effects were calculated within each cohort as Hedges′ *g* with sampling variance. Because GPL3921 and GPL571 originated from GSE14520, their estimates were first combined by inverse‐variance fixed‐effect weighting to give GSE14520 one contribution. TCGA‐LIHC, ICGC LIRI‐JP, and combined GSE14520 estimates were then synthesized with a DerSimonian–Laird random‐effects model [35]. The same hierarchy was used for YTHDC1 RNA abundance, the transferred hepatocyte state, and each specificity branch; CPTAC protein was displayed separately. Leave‐one‐study‐out analysis omitted each study separately. Overall survival was analyzed in TCGA‐LIHC (365 patients and 131 deaths), ICGC LIRI‐JP (231 and 42), GSE14520‐GPL3921 (221 and 85), and GSE14520‐GPL571 (21 and 11). Recurrence‐free survival was available for both GSE14520 strata (221 patients/121 events and 21/15). TCGA and ICGC follow‐up was converted from days to months by division by 30.4375, and TCGA endpoints followed the curated Pan‐Cancer Clinical Data Resource [22]. Within each stratum, continuous standardized YTHDC1 and state scores were entered into Cox models using survival 3.8.6. GSE14520 platform estimates were combined within the study before random‐effects pooling with the other cohorts. Adjusted models used stage, age, and sex where complete data and event counts permitted. Proportional hazards assumptions were checked with Schoenfeld residuals. Kaplan–Meier plots used median groups for visualization only.

### 2.11. ICI Cohort Processing and Response Analysis

Pretreatment tumor expression and patient‐level response labels were reconciled for GSE202069, GSE215011, and GSE279750, giving 37 nonduplicated patients: 8 responders and 9 nonresponders, 5 and 5, and 6 and 4, respectively. GSE202069 was processed from TPM, whereas GSE215011 and GSE279750 were processed from FPKM; each cohort was transformed as log2 (source abundance +1) and then standardized within cohort. Normalized or log‐transformed duplicate rows were not summed. Instead, every prespecified target gene was required to map to exactly one source row; all three cohorts passed this check with zero duplicate target rows. Primary features were YTHDC1, CD274, CD8A, and the CXCL9–CXCL10 mean. Prespecified programs were IFNG response (IFNG, CXCL9, CXCL10, STAT1, and IRF1), T‐cell inflamed (CD8A, IFNG, GZMB, CXCL9, CXCL10, CD274, and STAT1), antigen presentation (HLA‐A, HLA‐B, HLA‐C, B2M, TAP1, and TAP2), and myeloid context (CCL2 and CSF1). Responder‐minus‐nonresponder Hedges′ *g* values were calculated within cohort and pooled by random‐effects analysis. Exact label permutation was used for all response assignments when the number of combinations was at most 20,000 and 20,000 permutations with seed 20260717 otherwise. Leave‐one‐cohort‐out estimates, leave‐one‐patient‐out influence, program effects, and YTHDC1–program Spearman′s correlations were examined. No classifier, response threshold, or predictive performance metric was fitted.

### 2.12. Exploratory Single‐Cell Network Virtual Knockout

Exploratory network perturbation was conducted with scTenifoldKnk 1.0.3, which estimates differential regulation after an in silico gene knockout in single‐cell gene regulatory networks [36]. Raw counts from tumor sample hepatocytes were analyzed independently in GSE149614 and GSE290925. Eligibility required at least 50 cells per patient; GSE290925 patient HCC07 was excluded because only 22 eligible cells were available. Sixty cells per eligible patient were sampled, giving 600 cells from 10 patients in GSE149614 and 660 cells from 11 patients in GSE290925. The input was restricted to 1199 genes shared between cohorts. Counts were transformed to counts per million, and 20 networks were generated per cohort with makeNetworks using *q* = 0.9, 500 cells per network, three principal components, tensor rank 3, and strict directionality. For the virtual knockout, the outgoing YTHDC1 row was set to zero before manifold alignment and differential regulation ranking. The entire network subsampling analysis was repeated 20 times per cohort. Cross‐cohort evaluation used overlap among top‐ranked genes, Spearman′s rank correlation across all shared genes, pathway enrichment, and program‐level concordance. Leave‐one‐patient‐out stability used three networks per held‐out patient. Five expression‐, detection‐, and network degree–matched genes per cohort provided a comparator distribution. The outputs quantify network perturbation magnitude and reproducibility; they are neither measured knockout transcriptomes nor estimates of expression direction or causal effect.

### 2.13. Clinical Specimens and Ethical Approval

Paired HCC and adjacent tissue specimens were collected at the Second Affiliated Hospital of Chongqing Medical University with approval from the institutional Ethics Committee (Approval No. 157, 2020). The study included 20 paired specimens for YTHDC1 mRNA measurement, five pairs for Western blotting, and 20 pairs for immunohistochemistry. Tumor and adjacent tissues were analyzed as matched pairs, and numerical analyses used the corresponding source data.

### 2.14. Quantitative Real‐Time PCR

Total RNA from paired clinical tissues and transfected HCC cells was analyzed by quantitative real‐time PCR. Relative YTHDC1 abundance in the clinical samples was calculated with the 2^−*Δ* *C* *t*^ method. The tissue comparison included 20 paired HCC and adjacent specimens. Cell line assays compared negative‐control siRNA (siNC) with two independent YTHDC1‐targeting siRNAs (siYTHDC1#1 and siYTHDC1#2) in Huh‐7 and MHCC‐97H cells, with three replicates reported per condition.

### 2.15. Western Blotting

Proteins were extracted from clinical tissues or transfected cells in RIPA lysis buffer containing protease inhibitors, separated by electrophoresis, and transferred to polyvinylidene difluoride membranes. Membranes were incubated with the primary antibody overnight at 4°C and with the secondary antibody for 1 h at room temperature. Protein bands were detected by enhanced chemiluminescence and quantified with ImageJ, with GAPDH used as the loading control. The clinical assay included five matched HCC–adjacent pairs. The cell line assay compared siNC, siYTHDC1#1, and siYTHDC1#2 in both Huh‐7 and MHCC‐97H cells, with *n* = 3 reported for the knockdown experiment.

### 2.16. Immunohistochemistry

Paraffin‐embedded clinical sections underwent graded dehydration and heat‐induced antigen retrieval in sodium citrate solution. Nonspecific binding was blocked with goat serum for 1 h. Sections were then incubated with the primary antibody for 24 h at 4°C, washed three times with PBS, incubated with the secondary antibody, and developed with DAB. Staining was examined with an upright microscope. The analysis included staining scores from 20 paired HCC and adjacent specimens together with representative low‐ and high‐magnification fields from the original tissue panel.

### 2.17. Cell Culture and siRNA Transfection

Huh‐7 and MHCC‐97H HCC cells were used for perturbation experiments; Huh‐7 cells were obtained from Procell Life Science & Technology Co., Ltd. At approximately 30%–40% confluence, cells were transfected with siNC, siYTHDC1#1, or siYTHDC1#2. Knockdown efficiency was evaluated 3 days later by quantitative real‐time PCR and Western blotting before the proliferation and coculture assays were interpreted.

### 2.18. CCK‐8 Assay

HCC cells were seeded in 96‐well plates, and CCK‐8 solution was added at a 1: 25 dilution in culture medium. Over the 72‐h observation interval, absorbance was measured at 450 nm at 0, 24, 48, and 72 h for siNC, siYTHDC1#1, and siYTHDC1#2 in Huh‐7 and MHCC‐97H cells. Three replicates were reported per condition.

### 2.19. EdU Incorporation Assay

HCC cells were seeded in six‐well plates and incubated with 10 *μ*M EdU working solution for 6 h. Cells were harvested, washed with PBS, fixed in 4% paraformaldehyde for 15 min at room temperature, permeabilized with 0.3% Triton X‐100 for 20 min, and stained with the Click‐iT reaction cocktail. Samples were analyzed by flow cytometry, and the EdU‐positive fraction was used as the DNA synthesis readout for siNC, siYTHDC1#1, and siYTHDC1#2. Three replicates were reported per condition.

### 2.20. THP‐1 Differentiation and Transwell Migration Assay

THP‐1 cells were differentiated to a macrophage‐like state by PMA exposure. This model is widely used, although it does not fully reproduce the properties of primary macrophages [37]. After activation and adherence, THP‐1 cells were placed in the upper compartment of an 8‐*μ*m‐pore Transwell insert. The lower compartment contained Huh‐7 or MHCC‐97H cells transfected with siNC, siYTHDC1#1, or siYTHDC1#2. Migrated THP‐1‐derived cells were examined and counted in 200× fields, with three replicates reported per condition.

### 2.21. Statistical Analysis

Analyses were performed in R 4.5.1 and Python 3.13.9 unless another version is specified. Paired continuous measurements were assessed with two‐sided Wilcoxon signed‐rank tests, unpaired groups with two‐sided Wilcoxon rank‐sum tests, and associations with Spearman′s correlation. Effect sizes are reported with 95% confidence intervals, survival with Cox hazard ratios (HRs), and multiple testing with Benjamini–Hochberg‐adjusted *p* values where indicated. All tests were two‐sided, and *p* < 0.05 was considered statistically significant.

## 3. Results

### 3.1. Multicohort Profiling Establishes the Tissue‐Level Abundance Pattern of YTHDC1

The paired‐data evidence matrix confirmed five tumor–adjacent measurement strata spanning RNA and protein data (Figure 1A). In TCGA‐LIHC, YTHDC1 RNA was higher in 50 paired tumors (Hedges′ *g* = 0.371, 95% CI 0.084–0.657; paired Wilcoxon *p* = 0.016), and ICGC LIRI‐JP showed a smaller increase across 194 pairs (*g* = 0.156, 95% CI 0.014–0.297; *p* = 0.012) (Figure 1B,C). The GSE14520 strata differed: GPL3921 showed lower tumor expression in 214 pairs (*g* = −0.461, 95% CI −0.602 to −0.320; *p* = 6.89 × 10^−11^), whereas GPL571 provided an imprecise estimate across 19 pairs (*g* = −0.291, 95% CI −0.751 to 0.168; *p* = 0.352) (Figure 1D,E). CPTAC displayed a clear protein increase in 160 pairs (*g* = 1.214, 95% CI 1.009–1.418; *p* = 8.39 × 10^−25^) (Figure 1F,G). After the two GSE14520 platforms were combined within the study, the RNA random‐effects estimate was *g* = 0.016 (95% CI −0.471 to 0.504; *I* ^2^ = 95.9*%*), and leave‐one‐study‐out estimates reflected this heterogeneity (Figure 1H,I). Probe‐level sensitivity reproduced the platform difference (Figure 1J). Clinicopathological analysis linked higher YTHDC1 with advanced stage in TCGA (*g* = 0.461, *p* = 3.34 × 10^−4^) and showed smaller associations with grade, age, and sex in TCGA and ICGC (Figure 1K).

**Figure 1:**
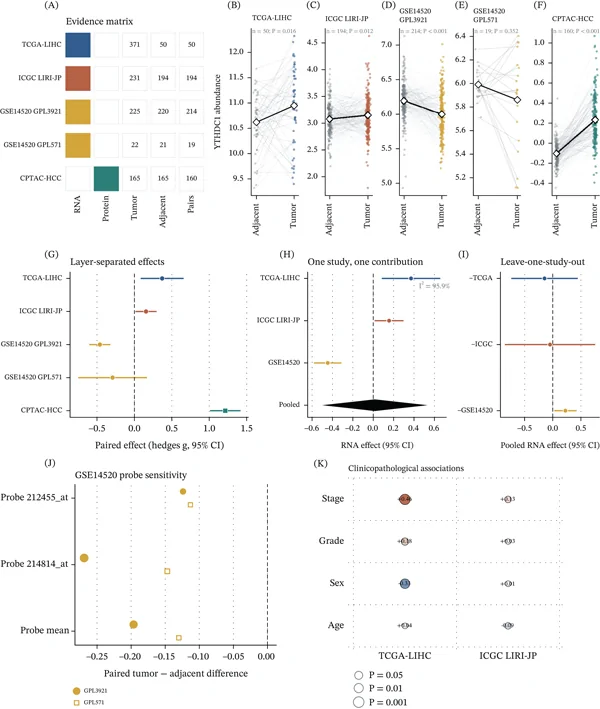
Multicohort paired RNA and protein evidence for YTHDC1 abundance in HCC. (A) Evidence matrix showing cohort, molecular layer, sample count, pairing, and primary source. Paired tumor and adjacent tissue YTHDC1 values in (B) TCGA‐LIHC, (C) ICGC LIRI‐JP, (D) GSE14520‐GPL3921, (E) GSE14520‐GPL571, and (F) CPTAC HCC protein data. Lines connect samples from the same patient. (G) Cohort‐specific paired Hedges′ *g* estimates with 95% CIs, with the protein layer displayed separately. (H) Study‐aware random‐effects synthesis of RNA estimates after combining the two GSE14520 platforms within the study. (I) Leave‐one‐study‐out RNA estimates. (J) GSE14520 sensitivity analysis for probes 212455_at, 214814_at, and their mean on each platform. (K) Associations of YTHDC1 with stage, grade, sex, and age in TCGA‐LIHC and ICGC LIRI‐JP. Point size in association panels reflects the number of patients.

### 3.2. Single‐Cell Atlases Map YTHDC1 Across Hepatic and Immune Compartments

Quality control retained 249,080 cells: 71,547 of 71,915 from GSE149614, 142,735 of 149,021 from GSE290925, and 34,798 of 49,674 from GSE112271 (Figure 2A,B). A cohort‐centered 38,281‐cell atlas constructed from 427 shared genes separated hepatocyte, T/NK, B, myeloid, endothelial, fibroblast, and other compartments, with canonical markers supporting their annotations (Figure 2C,D). Tissue, cohort, and patient overlays showed that the atlas incorporated all three datasets while preserving patient composition (Figure 2E–G). YTHDC1 was detected across hepatic and immune compartments, with differences in both normalized abundance and detection fraction among cell classes and patients (Figure 2H–J). In the eight GSE149614 patients with paired tissue hepatocytes, tumor–adjacent YTHDC1 summaries varied among patients (Figure 2K). Cross‐dataset comparison of tumor sample hepatocytes with nonhepatocyte compartments produced standardized effects of −0.726 in GSE149614 and −1.698 in GSE290925, whereas GSE112271 remained descriptive because it contained two patients. Five GSE149614 patients met the paired CNV sensitivity criteria; their patient‐level median tumor–normal difference was 0.0081 (*p* = 0.0625), providing a conditional check on the tumor sample hepatocyte definition (Figure 2L,M).

**Figure 2:**
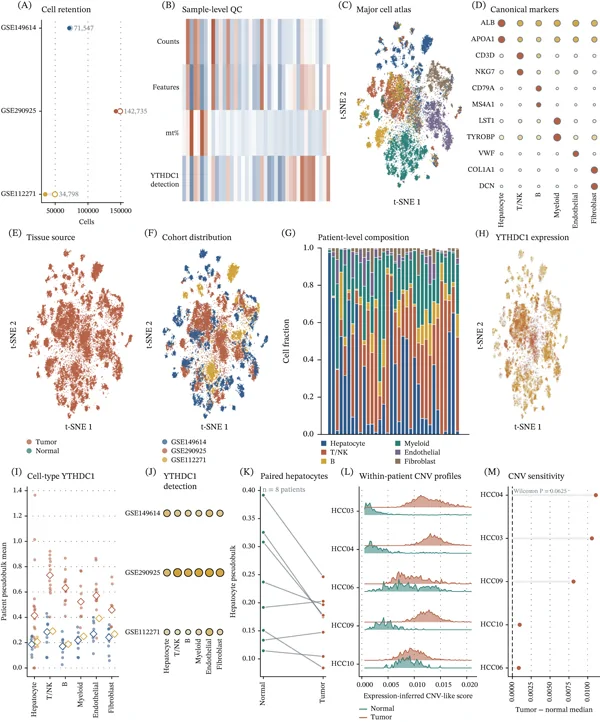
Single‐cell distribution of YTHDC1 across HCC compartments and CNV sensitivity analysis. (A) Cell‐retention summary for the three single‐cell datasets. (B) Specimen‐ and patient‐level quality control summaries. (C) Cohort‐centered t‐SNE atlas of the balanced 38,281‐cell visualization subset based on 427 shared genes. (D) Canonical marker support for the harmonized cell classes; dot size represents detection fraction, and color represents mean normalized expression. Atlas colored by (E) tissue source, (F) cohort, and (G) patient composition. (H) YTHDC1 expression on the atlas. (I) Patient‐level YTHDC1 abundance across cell classes. (J) YTHDC1 detection fractions by cell class and cohort. (K) Paired tumor–adjacent hepatocyte summaries in the eight eligible GSE149614 patients, together with cross‐dataset standardized comparisons of tumor sample hepatocytes and other compartments. (L) Within‐patient expression‐inferred CNV profiles in normal and tumor sample hepatocytes from five eligible GSE149614 patients. (M) Patient‐level CNV score differences and the fraction of tumor‐sample cells above the corresponding normal‐tissue 95th percentile. CNV results are sensitivity evidence for compartment definition.

### 3.3. Patient‐Blocked Analyses Resolve Depth‐Sensitive Hepatocyte Programs

Depth‐residualized YTHDC1 was defined within‐patient high and low states, and raw counts were aggregated into paired pseudobulk blocks for 10 GSE149614 and 11 GSE290925 patients (Figure 3A,B). The patient‐blocked edgeR models tested 18,159 genes in GSE149614 and 20,284 genes in GSE290925; one and 89 genes, respectively, met false − discovery − rate < 0.05 and absolute log_2_ fold − change ≥ 0.5, and the cross‐cohort gene effects showed modest concordance (Figure 3C–F). Competitive enrichment placed the ranked effects in Hallmark, Reactome, and Gene Ontology contexts and showed which pathway ranks were stable under leave‐one‐patient‐block‐out analysis (Figure 3G,H,K). Before depth adjustment, YTHDC1‐high hepatocytes had higher proliferation, E2F, and MYC program scores in both cohorts: The standardized effects were 1.141, 1.004, and 1.325 in GSE149614 and 0.904, 1.139, and 3.392 in GSE290925 (Figure 3I). Residualization for total counts, detected genes, and mitochondrial percentage shifted these estimates to 0.184, 0.205, and −0.037 in GSE149614 and −0.723, −0.709, and −0.946 in GSE290925 (Figure 3J), identifying library complexity as an important component of the raw program relationship.

**Figure 3:**
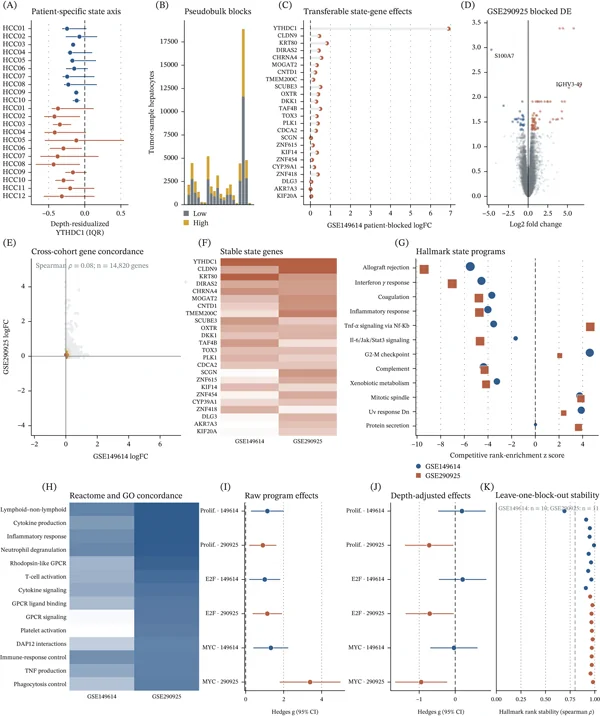
Patient‐blocked hepatocyte programs associated with YTHDC1. (A) Depth‐residualized YTHDC1 distributions used for within‐patient state assignment. (B) Paired pseudobulk design for GSE149614 and GSE290925. (C) Effect estimates for the 24 transferable genes in GSE149614. (D) Genome‐wide differential expression results in GSE290925. (E) Cross‐cohort agreement of gene‐level effects. (F) Genes with stable direction across patient blocks and cohorts. Competitive rank‐enrichment results for (G) Hallmark and (H) Reactome/Gene Ontology sets. (I) Raw patient‐level effects for proliferation, E2F, and MYC programs. (J) Corresponding effects after residualization for total counts, detected genes, and mitochondrial percentage. (K) Leave‐one‐patient‐block‐out stability of Hallmark pathway ranks. Effect estimates use the patient as the inferential unit.

### 3.4. Immune Cell Programs Place YTHDC1 Within a Patient‐Specific Immune Context

The integrated immune atlas resolved T/NK, B, and myeloid compartments and displayed marker support, tissue distribution, and patient composition across GSE149614 and GSE290925 (Figure 4A–D). YTHDC1 abundance and detection varied among immune compartments (Figure 4E), whereas cytotoxic, activation, inflammatory, interferon, and suppressive programs distinguished functional states within the atlas (Figure 4F,G). At the patient level, hepatocyte YTHDC1 was compared with immune cell abundance and immune program scores rather than individual cell observations (Figure 4H,I). The clearest nominal program associations were a positive correlation with the inflammatory score in GSE149614 (Spearman′s *ρ* = 0.661, *p* = 0.038) and an inverse correlation with the interferon score in GSE290925 (*ρ* = −0.713, *p* = 0.009). The full directional summary showed a mixture of concordant and cohort‐specific relationships across abundance and functional readouts (Figure 4J), indicating that YTHDC1 is situated within heterogeneous patient immune states.

**Figure 4:**
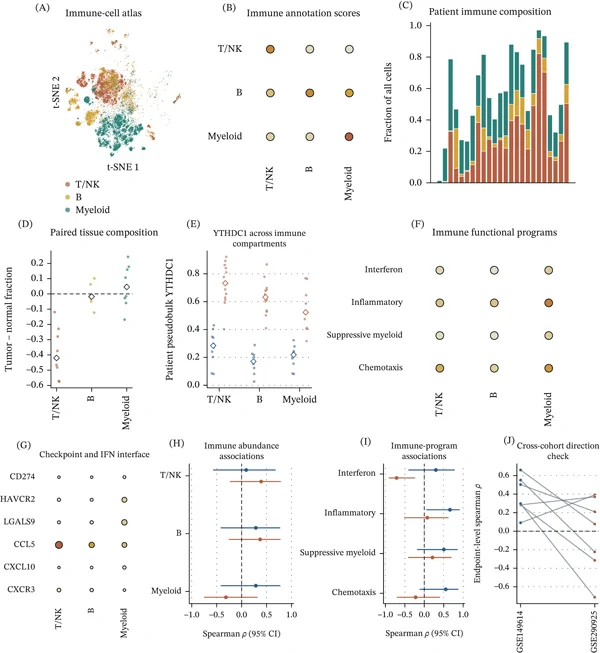
Lymphoid and immune context mapping of YTHDC1 in HCC. (A) Integrated immune cell atlas comprising T/NK, B, and myeloid compartments. (B) Annotation score support for the immune compartments. (C) Patient‐level immune cell composition. (D) Paired tissue differences in immune composition where matched samples were available. (E) YTHDC1 abundance and detection across immune compartments. (F) Cytotoxic, activation, inflammatory, interferon, and suppressive program scores. (G) Expression of selected immune checkpoint and interferon interface genes. (H) Patient‐level associations between hepatocyte YTHDC1 and immune cell abundance. (I) Associations between hepatocyte YTHDC1 and immune program scores. (J) Cross‐cohort direction of the displayed associations. Points represent patient‐level summaries, and horizontal lines show 95% CIs where estimable.

### 3.5. Myeloid State Mapping Identifies Reproducible Ecological Components

Myeloid cells separated into C1QC/FOLR2, FCN1 monocyte, inflammatory, interferon, and SPP1/TREM2 states, with canonical genes supporting each annotation (Figure 5A,B). Their relative abundance differed across patients and between paired tissue contexts, and YTHDC1 expression varied across the five states (Figure 5C–E). Chemotaxis, suppressive myeloid, inflammatory, and interferon program summaries, together with SPP1/TREM2‐related expression, described distinct patient‐level myeloid ecologies (Figure 5F,G). Associations between hepatocyte YTHDC1 and myeloid readouts were modest after false‐discovery rate adjustment: For example, the chemotaxis correlation was 0.406 in GSE149614 and −0.427 in GSE290925, and the suppressive myeloid correlation in GSE290925 was −0.566; their displayed adjusted *p* values were 0.638 (Figure 5H). Cross‐cohort directional comparison and varying the minimum‐cell threshold showed which associations were stable to analysis choices (Figure 5I,J). These results establish the distribution and patient heterogeneity of the myeloid states used in subsequent hepatocyte‐to‐myeloid analysis.

**Figure 5:**
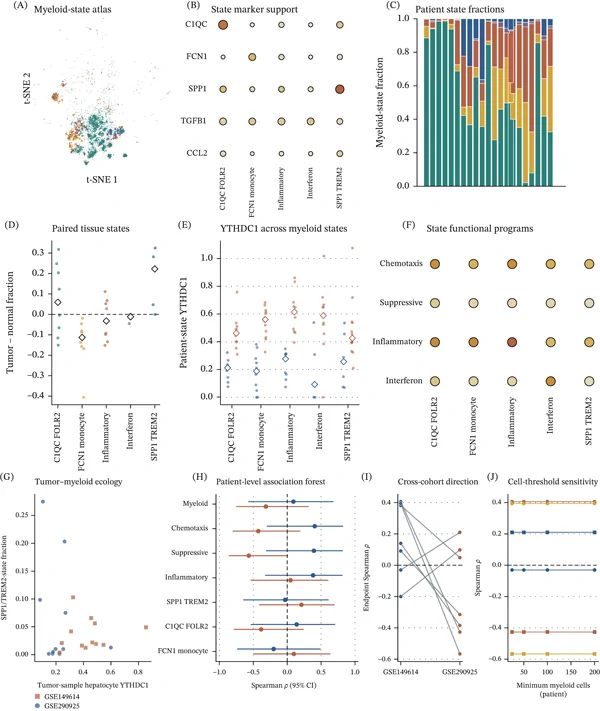
Patient‐level myeloid states associated with the YTHDC1 context. (A) Integrated myeloid cell atlas showing C1QC/FOLR2, FCN1 monocyte, inflammatory, interferon, and SPP1/TREM2 states. (B) Marker support for state annotation. (C) Patient‐level state fractions. (D) Paired tissue comparison of myeloid state composition. (E) YTHDC1 abundance across myeloid states. (F) Chemotaxis, suppressive myeloid, inflammatory, and interferon program summaries. (G) SPP1/TREM2 state ecology across patients. (H) Cohort‐specific associations between hepatocyte YTHDC1 and myeloid fractions or programs. (I) Directional concordance across GSE149614 and GSE290925. (J) Sensitivity of selected associations to the minimum myeloid cell threshold. Displayed false‐discovery rates were calculated within the myeloid association family.

### 3.6. Cross‐Cohort Expression Support Prioritizes the SPP1–CD44 Axis

All 12 prespecified hepatocyte‐to‐myeloid ligand–receptor axes were evaluated in parallel across GSE149614 and GSE290925 (Figure 6A). SPP1–CD44 received the highest normalized cross‐cohort priority score (1.000), followed by TGFB1–TGFBR1/TGFBR2 (0.244), CSF1–CSF1R (0.160), CXCL8–CXCR2 (0.139), and CXCL12–CXCR4 (0.137) (Figure 6B). Patient‐level ligand and receptor summaries supported the ranking and showed the contribution of each cellular compartment (Figure 6C,D). SPP1–CD44 expression support and the other leading axes varied among patients, and their relationships with hepatocyte YTHDC1 and receiver cell programs were examined in separate correlation families (Figure 6E–I). No YTHDC1–axis or axis–program association passed the Benjamini–Hochberg correction; the smallest adjusted value was *q* = 0.227. Leave‐one‐patient‐out analysis retained nearly identical axis rankings across 22 patients (Spearman′s *ρ* = 0.979 − 1.000; Figure 6J). Thus, SPP1–CD44 was the most stable expression‐supported candidate axis in this prespecified screen, which ranked coexpression support rather than signaling activity.

**Figure 6:**
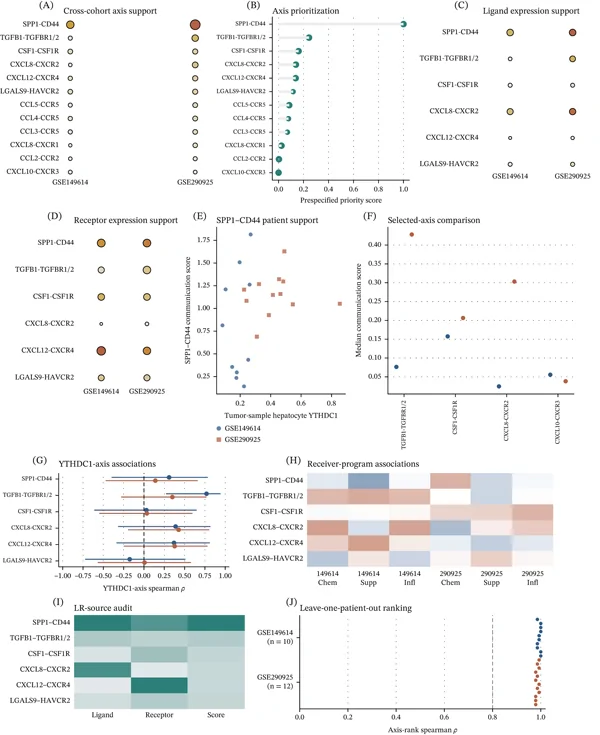
Cross‐cohort expression support for hepatocyte‐to‐myeloid ligand–receptor axes. (A) Expression‐support matrix for 12 prespecified axes in GSE149614 and GSE290925. (B) Cross‐cohort priority scores. (C) Patient‐level ligand expression in tumor sample hepatocytes. (D) Receptor expression in myeloid cells. (E) Patient‐level support for SPP1–CD44. (F) Comparison of the leading axes across cohorts. (G) Associations between hepatocyte YTHDC1 and axis support. (H) Associations between axis support and receiver cell programs. (I) Combined expression and database membership audit. (J) Leave‐one‐patient‐out stability of axis rankings across 22 patients. The analysis evaluates expression support and does not estimate communication probability.

### 3.7. A Transferred Hepatocyte State Captures a Proliferation‐Linked Tissue Signal

Twenty‐four genes with a shared positive direction between YTHDC1‐high and YTHDC1‐low hepatocytes were carried forward from the two single‐cell cohorts (Figure 7A–C). Gene availability auditing supported projection across TCGA‐LIHC, ICGC LIRI‐JP, and both GSE14520 platforms (Figure 7D). In paired tissues, the state was generally higher in tumors across the RNA cohorts (Figure 7E–G); after the GSE14520 platforms were combined within the study, the random‐effects estimate was *g* = 0.418 (95% CI 0.039–0.797; *I* ^2^ = 92.7*%*) (Figure 7H). The estimate remained positive after removal of YTHDC1 (*g* = 0.450, 95% CI 0.097–0.804) and shifted after removal of the four proliferation‐linked genes (*g* = −0.238, 95% CI −0.857 to 0.381); the fixed comparator produced a larger tissue effect (*g* = 1.381, 95% CI 1.087–1.675) (Figure 7I,J). These branch analyses show that the transferred state captures a broader tissue and proliferation‐associated program rather than a single‐gene readout.

**Figure 7:**
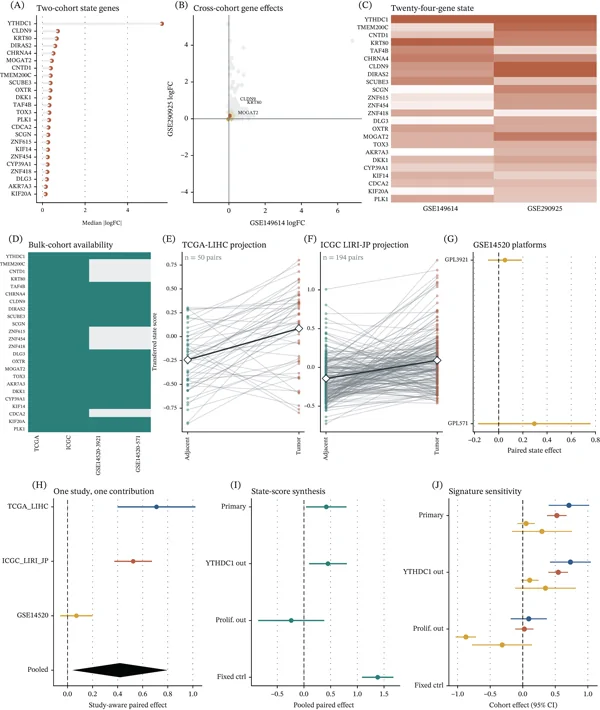
Transfer of the YTHDC1‐high hepatocyte state to paired bulk cohorts. (A) Composition of the 24‐gene state. (B) Cohort‐specific single‐cell effect estimates for state genes. (C) Cross‐cohort heatmap of transferable‐gene effects. (D) Gene availability audit for TCGA‐LIHC, ICGC LIRI‐JP, GSE14520‐GPL3921, and GSE14520‐GPL571. Paired tumor–adjacent state scores in (E) TCGA‐LIHC, (F) ICGC LIRI‐JP, and (G) the two GSE14520 platforms. (H) Study‐specific paired effects and random‐effects synthesis after within‐study combination of the GSE14520 platforms. (I) Leave‐one‐study‐out estimates. (J) Specificity branches removing YTHDC1, removing four proliferation‐linked genes, and applying a fixed comparator set. The state was defined before bulk projection and was not optimized against tissue class or outcome.

### 3.8. Study‐Aware Outcome Analysis Links YTHDC1 With Overall Survival

Four strata contributed overall‐survival data: TCGA‐LIHC (365 patients/131 deaths), ICGC LIRI‐JP (231/42), GSE14520‐GPL3921 (221/85), and GSE14520‐GPL571 (21/11) (Figure 8A). Kaplan–Meier displays provided cohort‐level visualization of continuous‐model patterns (Figure 8B–D). Study‐aware univariable Cox analysis gave a pooled HR of 1.202 per 1‐standard‐deviation increase in YTHDC1 (95% CI 1.062–1.361; *I* ^2^ = 0), and leave‐one‐study‐out HRs ranged from 1.157 to 1.234 (Figure 8E–G). The transferred state, YTHDC1‐removed state, proliferation‐removed state, and fixed comparator had pooled HRs of 1.303, 1.304, 1.117, and 1.414, respectively. In TCGA, the univariable YTHDC1 association (HR 1.248, 95% CI 1.047–1.486) attenuated after adjustment for stage, age, and sex (HR 1.127, 95% CI 0.934–1.360); ICGC estimates were 1.020 and 0.956 (Figure 8H). Proportional hazards diagnostics identified a TCGA YTHDC1 signal (*p* = 0.021), and recurrence‐free survival analyses in the two GSE14520 strata provided complementary endpoint estimates (Figure 8I,J).

**Figure 8:**
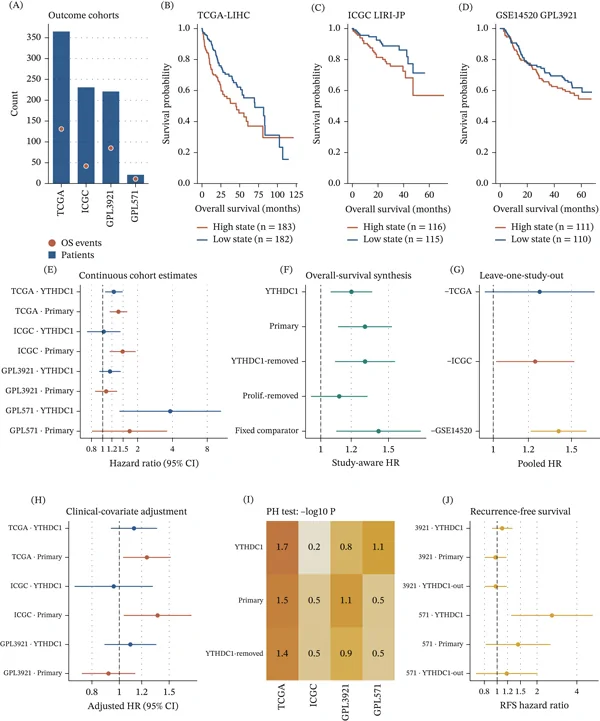
Study‐aware clinical outcome associations of YTHDC1 and transferred states. (A) Cohort manifest with sample size and event count. Kaplan–Meier displays for (B) TCGA‐LIHC, (C) ICGC LIRI‐JP, and (D) GSE14520‐GPL3921, grouped at the within‐cohort median for visualization. (E) Cohort‐specific continuous Cox estimates for YTHDC1. (F) Study‐aware random‐effects estimates for YTHDC1, the transferred state, the YTHDC1‐removed state, the proliferation‐removed state, and the fixed comparator. (G) Leave‐one‐study‐out YTHDC1 estimates. (H) Univariable and clinically adjusted YTHDC1 estimates in cohorts with sufficient data. (I) Proportional hazards diagnostics. (J) Recurrence‐free survival estimates in the GSE14520 platform. Points show HRs per 1‐standard‐deviation increase, and horizontal lines show 95% CIs.

### 3.9. ICI Cohorts Define the Response‐Associated Range of YTHDC1 and Immune Programs

The three pretreatment ICI cohorts comprised 37 patients: GSE202069 included 8 responders and 9 nonresponders, GSE215011 included 5 and 5, and GSE279750 included 6 and 4 (Figure 9A). Cohort‐standardized patient features displayed the within‐study distributions of YTHDC1, CD274, CD8A, and CXCL9–CXCL10 (Figure 9B–E). The pooled responder‐minus‐nonresponder effect for YTHDC1 was *g* = 0.055 (95% CI −0.600 to 0.710; *p* = 0.870; *I* ^2^ = 0), and leave‐one‐cohort‐out and patient influence analyses showed the uncertainty associated with the sample size (Figure 9F,G,I). CD274 had a pooled effect of 0.601 (95% CI −0.067 to 1.270), whereas the IFNG response, T‐cell‐inflamed, antigen presentation, and myeloid context programs had pooled effects of 0.318, 0.379, 0.627, and 0.513, respectively (Figure 9H,J). YTHDC1–program correlations varied by cohort; the largest displayed value was the association with the T‐cell‐inflamed score in GSE279750 (*ρ* = 0.721, *p* = 0.019) (Figure 9K). These estimates characterize the observed response context and its uncertainty without defining a predictive threshold.

**Figure 9:**
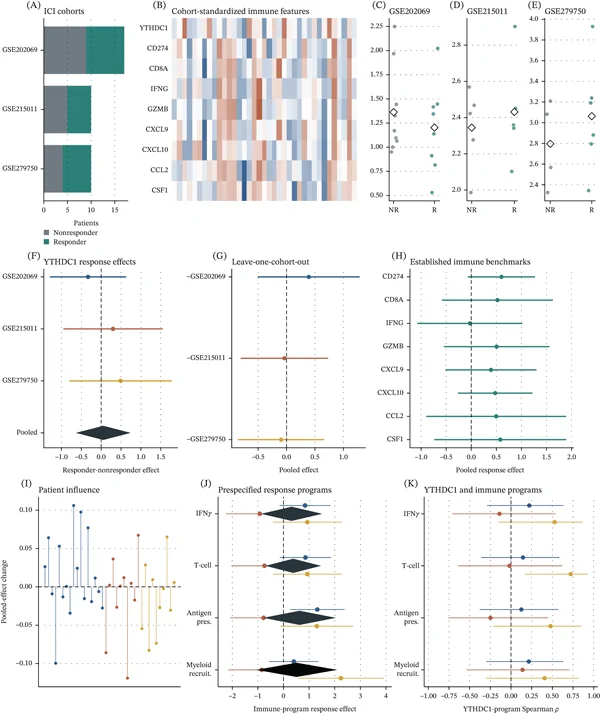
YTHDC1 and immune program estimates in ICI‐treated HCC cohorts. (A) Responder and nonresponder counts in GSE202069, GSE215011, and GSE279750. (B) Standardized patient‐level YTHDC1 and immune features by cohort. YTHDC1 distributions according to response in (C) GSE202069, (D) GSE215011, and (E) GSE279750. (F) Cohort‐specific and pooled responder‐minus‐nonresponder YTHDC1 effects. (G) Leave‐one‐cohort‐out estimates. (H) Comparative effects for YTHDC1, CD274, CD8A, and CXCL9/CXCL10. (I) Leave‐one‐patient‐out influence on the pooled YTHDC1 estimate. (J) Cohort‐specific and pooled effects for the IFNG response, T‐cell‐inflamed, antigen presentation, and myeloid context programs. (K) Cohort‐specific correlations between YTHDC1 and the four immune programs. No response classifier or threshold was fitted.

### 3.10. Network Virtual Knockout Characterizes Cross‐Cohort Perturbation Concordance

The exploratory virtual knockout analysis included 600 tumor sample hepatocytes from 10 GSE149614 patients and 660 cells from 11 GSE290925 patients, with 1199 shared genes and repeated network subsampling (Figure 10A). The wild‐type YTHDC1 ego‐networks and cohort‐specific differential regulation ranks showed distinct local structures (Figure 10B,C,E,F). Six genes in GSE149614 and three in GSE290925 reached a false‐discovery rate below 0.05, and cohort‐specific ranked‐gene enrichment generated pathway‐level summaries (Figure 10D,G). Cross‐cohort overlap was one gene among the Top 25 (Jaccard index 0.020, two‐sided Fisher exact *p* = 0.413), five among the Top 50 (Jaccard index 0.053, *p* = 0.053), and 27 among the Top 10% (Jaccard index 0.127, odds ratio 3.074, *p* = 1.61 × 10^−5^) (Figure 10H). Across all 1199 genes, perturbation ranks were positively correlated (*ρ* = 0.238, 95% CI 0.182–0.292; *p* = 8.57 × 10^−17^; Figure 10I), with additional pathway, program, repeated‐network, and leave‐one‐patient‐out comparisons shown in Figure 10J,K. Matched expression, detection, and network degree comparators placed the YTHDC1 perturbation magnitude within the observed comparator range in both cohorts (Figure 10L). The result, therefore, describes reproducible network‐ranking structure and perturbation magnitude, not a measured knockout transcriptome, expression direction, or causal consequence.

**Figure 10:**
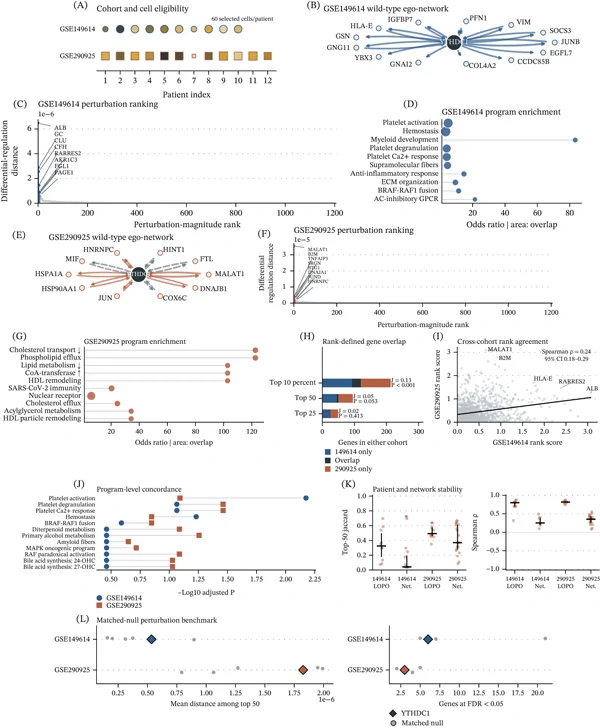
Exploratory YTHDC1 network virtual knockout in tumor sample hepatocytes. (A) Eligibility, patient representation, cell sampling, and shared‐feature summary for GSE149614 and GSE290925. Wild‐type YTHDC1 ego‐networks in (B) GSE149614 and (E) GSE290925. Cohort‐specific differential regulation ranks after virtual YTHDC1 knockout. (D,G) Enrichment summaries derived from the ranked perturbation outputs. (H) Cross‐cohort overlap among the Top 25, Top 50, and Top 10% of ranked genes. (I) Spearman′s agreement of perturbation ranks across all 1199 shared genes. (J) Pathway‐ and program‐level concordance. (K) Repeated‐network and leave‐one‐patient‐out stability. (L) Perturbation magnitude relative to expression‐, detection‐, and network degree–matched comparator genes. The panels quantify network perturbation magnitude and reproducibility; they do not represent a measured knockout transcriptome, expression direction, or causal effect.

### 3.11. Paired Clinical Specimens Confirm Increased YTHDC1 Abundance in HCC

The preserved clinical figure connects public cohort evidence to independent tissue measurements (Figure 11). In the original public data panels, a paired *t*‐test showed higher ICGC RNA values in tumors from 194 patients (*p* = 0.031), and paired CPTAC protein values showed a larger increase across 160 patients (*p* < 0.001) (Figure 11A,B). In the clinical series, quantitative real‐time PCR showed increased YTHDC1 mRNA in 20 paired HCC and adjacent samples (Figure 11C). Western blotting in five matched pairs showed higher tumor protein abundance, with GAPDH as the loading control (Figure 11D). Immunohistochemistry in 20 paired specimens also showed higher tumor staining scores, supported by representative fields from three pairs and the original low‐ and high‐magnification tissue images (Figure 11E). The agreement between clinical qRT‐PCR, Western blotting, immunohistochemistry, and public proteomics provides tissue‐level support for increased YTHDC1 abundance in HCC.

**Figure 11:**
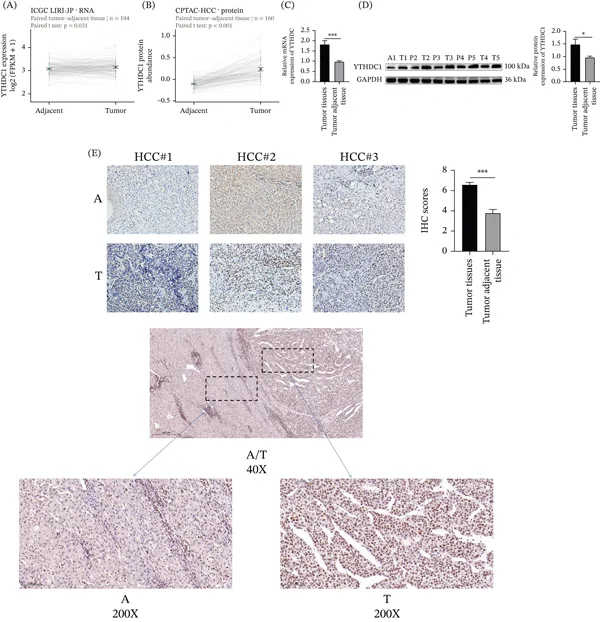
Public cohort and paired clinical tissue validation of YTHDC1 abundance. (A) Original paired *t*‐test display of YTHDC1 RNA abundance in 194 ICGC LIRI‐JP tumor and adjacent tissue samples. (B) Paired YTHDC1 protein abundance in 160 CPTAC HCC samples. (C) Quantitative real‐time PCR summary for 20 paired clinical HCC and adjacent tissues. (D) Original Western blots and densitometric summary for five matched tissue pairs, with GAPDH as the loading control. (E) Original immunohistochemistry panel with paired staining scores from 20 patients, representative adjacent and tumor fields from three cases, and the original low‐ and high‐magnification tissue images. Lines connect matched samples; diamonds and error bars summarize paired measurements. A denotes adjacent tissue, and T denotes tumor.  ^∗^ *p* < 0.05;  ^∗∗∗^ *p* < 0.001.

### 3.12. YTHDC1 Silencing Reduces HCC Cell Proliferation and THP‐1 Migration

Two independent siRNAs reduced YTHDC1 mRNA in Huh‐7 and MHCC‐97H cells compared with siNC, and Western blotting with densitometry confirmed lower protein abundance (Figure 12A,B). Across the 72‐h CCK‐8 time course, both YTHDC1‐targeting siRNAs produced lower absorbance than siNC in each cell line (Figure 12C). EdU flow cytometry profiles and the quantified EdU‐positive fractions showed reduced DNA synthesis after YTHDC1 silencing (Figure 12D). In the Transwell coculture assay, fewer THP‐1‐derived macrophage‐like cells migrated toward YTHDC1‐silenced Huh‐7 or MHCC‐97H cells, as shown by the original microscopic fields and quantification (Figure 12E). Concordant results from two siRNAs and two cell lines support a contribution of YTHDC1 to proliferation‐related phenotypes and tumor cell–dependent macrophage‐like cell migration in these experimental systems.

**Figure 12:**
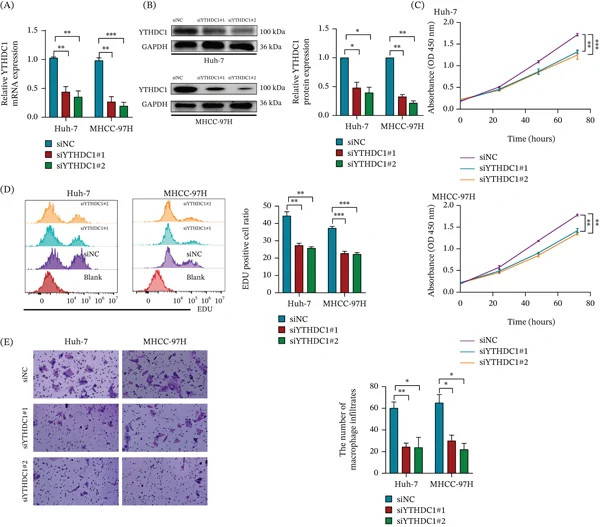
Functional consequences of YTHDC1 silencing in HCC cell models. (A) Quantitative real‐time PCR after transfection with siNC, siYTHDC1#1, or siYTHDC1#2 in Huh‐7 and MHCC‐97H cells. (B) Original Western blots and densitometric summaries of YTHDC1 knockdown, with GAPDH as the loading control. (C) CCK‐8 time courses at 0, 24, 48, and 72 h. (D) Original EdU flow cytometry distributions and reported EdU‐positive fractions. (E) Original Transwell fields and quantification of THP‐1‐derived macrophage‐like cells migrating toward control or YTHDC1‐silenced HCC cells at 200× magnification. Three replicates were reported per condition. ∗*p* < 0.05, ∗∗*p* < 0.01, and ∗∗∗*p* < 0.001, respectively.  ^∗^ *p* < 0.05;  ^∗∗^ *p* < 0.01;  ^∗∗∗^ *p* < 0.001.

## 4. Discussion

This study integrates paired bulk RNA, proteomics, single‐cell data, clinical outcomes, ICI‐treated cohorts, paired clinical specimens, and functional assays to characterize YTHDC1 in HCC. The main findings are that YTHDC1 protein abundance is consistently higher in paired tumors, YTHDC1 is distributed across hepatic and immune compartments, and its patient‐level context includes hepatocyte programs, myeloid states, and stable expression support for the SPP1–CD44 axis. A transferred hepatocyte state captured a tumor‐associated signal with a substantial proliferation component, and study‐aware survival analysis linked higher YTHDC1 abundance with poorer overall survival. The clinical specimens and cell experiments then supplied orthogonal tissue and perturbation evidence.

The tissue analysis illustrates why RNA and protein layers should be interpreted together. TCGA and ICGC showed modest increases in paired YTHDC1 RNA, whereas GSE14520 showed a platform‐consistent decrease and produced marked heterogeneity in the study‐aware RNA synthesis. In contrast, CPTAC and the clinical Western blot and immunohistochemistry measurements consistently supported higher protein abundance. This pattern is biologically plausible for an m6A reader involved in splicing and nuclear export [9, 10], because transcript abundance alone may not capture protein regulation. It is also consistent with prior work linking m6A regulators to HCC behavior and immune features [11, 12, 38]. The cross‐platform difference argues for protein‐aware validation when YTHDC1 is studied as a tissue biomarker [39].

Single‐cell mapping added the cellular resolution absent from bulk tissue profiles. YTHDC1 was present in hepatocytes and several immune compartments, whereas patient‐blocked analysis showed that raw proliferation, E2F, and MYC associations were strongly influenced by library complexity. The transferred 24‐gene state remained higher in tumor tissue after YTHDC1 was removed and changed substantially when four proliferation‐linked genes were removed. Together, these findings suggest that the state reflects a broader proliferative tissue program around YTHDC1 rather than a specific transcriptional output of YTHDC1. Patient‐level pseudobulk models and leave‐one‐patient‐block‐out analyses reduce cell number–driven precision inflation, an important issue in single‐cell differential expression [20].

The immune analyses placed YTHDC1 in a multicellular setting [40] without assuming that coexpression establishes signaling. Myeloid cells occupied C1QC/FOLR2, FCN1, inflammatory, interferon, and SPP1/TREM2 states, and SPP1–CD44 emerged as the most stable expression‐supported hepatocyte‐to‐myeloid axis. SPP1‐positive macrophages have been associated with immune suppression in HCC [41], and macrophage‐centered classifications have been proposed to describe clinical heterogeneity [42]. Our finding provides a reproducible candidate context for mechanistic investigation. The THP‐1 migration result adds functional support for a tumor cell–dependent migratory phenotype, although the responsible soluble or contact‐dependent mediator was not identified.

The clinical outcome results were modest yet consistent at the univariable study level. The pooled overall‐survival HR for YTHDC1 was 1.20 with no detected between‐study heterogeneity, and its direction was retained in leave‐one‐study‐out analyses. Adjustment attenuated the cohort‐specific estimates, and a proportional hazards signal was detected in TCGA. These results support YTHDC1 as an outcome‐associated continuous feature rather than a ready‐to‐use prognostic model [43]. This distinction is important in HCC, where stage, liver function, etiology, and treatment contribute strongly to prognosis [44]. Prospective evaluation with harmonized clinical covariates would be required before clinical use.

The ICI analysis provided a direct treatment‐related assessment across three cohorts. YTHDC1 itself showed a pooled responder–nonresponder effect close to zero, whereas CD274 and several immune programs had positive estimates with broad confidence intervals. The cohort‐specific YTHDC1–program correlations show that its immune context can vary across treatment datasets [45]. These observations fit the broader view that ICI response in HCC is governed by interacting tumor, immune, vascular, and etiologic factors rather than a single transcript [6, 46–50]. Accordingly, the present data support mechanistic study of YTHDC1 within immune programs, whereas treatment response prediction will require larger cohorts and predefined multivariable validation.

The network virtual knockout extended the analysis from observed expression to an exploratory perturbation of single‐cell regulatory networks. The modest positive correlation of cross‐cohort perturbation ranks and enrichment among the Top 10% of genes indicates a reproducible component, whereas the few false‐discovery‐rate‐significant genes and matched‐null results define its scale. scTenifoldKnk is designed to compare gene regulatory networks after an in silico perturbation [36]; its output is a differential regulation ranking, not a measured post–knockout expression profile. The experimental siRNA results, therefore, remain the direct perturbation evidence: In two HCC cell lines, two siRNAs reduced YTHDC1 abundance, CCK‐8 and EdU readouts, and THP‐1 migration.

Several boundaries apply to the integrated evidence. Public cohorts differed in etiology, platform, tissue annotation, treatment, biopsy timing, and response definition; The ICI analysis contained 37 patients. Tumor sample hepatocytes were defined by lineage and specimen context, and expression‐inferred CNV was available in five paired patients as a sensitivity analysis. GSE112271 represented two patients and was used descriptively. Single‐cell associations were observational, the ligand–receptor analysis measured expression support rather than communication, and the virtual knockout measured network perturbation ranks rather than causal expression changes. The clinical specimens came from one center, experimental sample sizes were modest, and PMA‐differentiated THP‐1 cells do not reproduce primary macrophage biology [37]. These considerations set the scope for interpretation while preserving the convergence of paired protein, clinical tissue, survival, and cell perturbation evidence.

## 5. Conclusion

YTHDC1 is increased at the protein level in HCC and is associated with cellular, immune, and outcome features across independent patient cohorts. Its silencing reduces proliferation‐related readouts and tumor cell–dependent macrophage‐like cell migration in the tested models, supporting further mechanistic and clinically harmonized evaluation.

## Author Contributions

Conceptualization, Siqi Luo, Shifeng Lou, and Lin Lv; methodology, Siqi Luo, Diguang Wen, and Jingyi Zhang; software, Siqi Luo, Diguang Wen, Yue Li, and Jiao Lu; validation, Siqi Luo, Diguang Wen, Jingyi Zhang, Yue Li, Jiao Lu, Zuojin Liu, Shifeng Lou, and Lin Lv; formal analysis, Jianchuan Deng, Hanqing Zeng, Yue Li, and Jiao Lu; investigation, Zuojin Liu, Shifeng Lou, and Lin Lv; resources, Jianchuan Deng, Hanqing Zeng, Zuojin Liu, Shifeng Lou, and Lin Lv; data curation, Siqi Luo and Diguang Wen; writing—original draft, Siqi Luo and Diguang Wen; writing—review and editing, Jingyi Zhang and Lin Lv; visualization, Jingyi Zhang, Yue Li, and Jiao Lu; supervision, Jianchuan Deng and Hanqing Zeng; project administration, Zuojin Liu, Shifeng Lou, and Lin Lv. All authors reviewed the results. Siqi Luo, Diguang Wen, and Jingyi Zhang contributed equally and share first authorship.

## Funding

This study was funded by the Chongqing Natural Science Foundation Innovation and Development Joint, CSTB2023NSCQ‐LZX0052, and the Chongqing Natural Science Foundation–Chongqing Science and Technology Development Foundation General Program, CSTB2024NSCQ‐KJFZMSX0060.

## Disclosure

All authors approved the final manuscript. An earlier version of this study was posted as a preprint (doi:10.21203/rs.3.rs-1546856/v1).

## Ethics Statement

Paired HCC and adjacent tissue specimens were collected at the Second Affiliated Hospital of Chongqing Medical University, and the study protocol was approved by the institutional Ethics Committee (Approval No. 157, 2020).

## Consent

All authors reviewed the manuscript and approved its publication.

## Conflicts of Interest

The authors declare no conflicts of interest.

## Data Availability

The public data analyzed in this study are available through TCGA/GDC, ICGC LIRI‐JP, CPTAC/PDC study PDC000198, and GEO accessions GSE14520, GSE149614, GSE290925, GSE112271, GSE202069, GSE215011, and GSE279750. The accession registry, processed analysis tables, figure records, and executable scripts are maintained with the study files. De‐identified clinical and laboratory source data are available from the corresponding authors subject to institutional approval and reasonable request.
